# Relation of anxiety and other psychometric measures, balance deficits, impaired quality of life, and perceived state of health to dizziness handicap inventory scores for patients with dizziness

**DOI:** 10.1186/s12955-020-01445-6

**Published:** 2020-06-26

**Authors:** D. A. Schmid, J. H. J. Allum, M. Sleptsova, A. Welge-Lüssen, R. Schaefert, G. Meinlschmidt, W. Langewitz

**Affiliations:** 1 Department of Psychosomatic Medicine, Faculty of Medicine, University of Basel and University Hospital Basel, Hebelstr 2, CH-4031 Basel, Switzerland; 2 Department of Psychosomatic Medicine, Cantonal Hospital St. Gallen, St. Gallen, Switzerland; 3 Department of ORL, Faculty of Medicine, University of Basel and University Hospital Basel, Basel, Switzerland; 4 Division of Clinical Psychology and Cognitive Behavioral Therapy, International Psychoanalytic University, Berlin, Germany; 5 Division of Clinical Psychology and Epidemiology, Department of Psychology, University of Basel, Basel, Switzerland

**Keywords:** Group cognitive behavioral therapy, Anxiety, Psychoeducation, Balance deficits, Dizziness, Vestibular rehabilitation

## Abstract

**Background:**

An important question influencing therapy for dizziness is whether the strengths of the relationships of emotional and functional aspects of dizziness to 1) anxiety and other mental states, 2) perceived state of health (SoH) and quality of life (QoL) are different in patients with and without normal balance control. We attempted to answer this question by examining these dimensions’ regression strengths with Dizziness Handicap Inventory (DHI) scores.

**Methods:**

We divided 40 patients receiving group cognitive behavioural therapy (CBT) and vestibular rehabilitation for dizziness, into 2 groups: dizziness only (DO) and normal balance control; dizziness and a quantified balance deficit (QBD). Group-wise, we first performed stepwise multivariate regression analysis relating total DHI scores with Brief Symptom Inventory (BSI) sub-scores obtained pre- and post-therapy. Then, regression analysis was expanded to include SoH, QoL, and balance scores. Finally, we performed regressions with DHI sub-scores.

**Results:**

In both groups, the BSI phobic anxiety state score was selected first in the multivariate regression analysis. In the DO group, obsessiveness/compulsiveness was also selected. The correlation coefficient, R, was 0.74 and 0.55 for the DO and QBD groups, respectively. When QoL and SoH scores were included, R values increased to 0.86 and 0.74, explaining in total 74, and 55% of the DHI variance for DO and QBD groups, respectively. Correlations with balance scores were not significant (R ≤ 0.21). The psychometric scores selected showed the strongest correlations with emotional DHI sub-scores, and perceived QoL and SoH scores with functional DHI sub-scores.

**Conclusions:**

Our findings suggest that reducing phobic anxiety and obsessiveness/compulsiveness during CBT may improve emotional aspects of dizziness and targeting perceived SoH and QoL may improve functional aspects of dizziness for those with and without normal balance control.

## Introduction

Dizziness and vertigo have a profound negative effect on the daily activities of 10% of those younger than 60 and 20% of those older than 60 years of age [1]. Accordingly, these two symptoms are among the most commonly occurring symptoms of patients examined by neurologists [2]. Considerable effort has been focussed on appropriate treatment plans for these symptoms (for a review see Dieterich and Staab [3]). One focus for patients with an identifiable structural medical disorder such as a unilateral peripheral vestibular deficit (UVD) is to use vestibular rehabilitation (VR) exercises to improve balance control [1, 4]. Others have suggested that patients with a fear of vertigo (a distorted sense of body motion) and dizziness (distorted spatial orientation) – see [3, 5, 6] for definitions of these symptoms – should be offered psychotherapeutic treatment [7–10]. For example, when patients with dizziness and vertigo (here collectively termed dizziness) who had normal balance control were provided CBT, the improvements in dizziness and avoidance behaviours lasted at least 6 months [7–10]. Yet a third approach is to combine CBT with VR. Using this approach, Yardley et al. [1] were able to show improvements in psychometric sub-scores thereby confirming the suggestions of others [3, 4] of the suitability of this treatment for both patients with and without an accompanying balance deficit [11]. In short, several authors have taken a multimodal approach to treating dizziness. For example Limburg and colleagues [12] investigated the effect of a multimodal approach as part of an inpatient treatment and found no significant change in anxiety, but small effects on depression, somatization and quality of life, at discharge, and at 6 months follow-up.

Apart from examining the association of psychometric scores on a standardized subjective assessment of the impact of dizziness on daily life using the dizziness handicap inventory (DHI, [13, 14]), other authors have examined the relationships of subjective assessments of quality of life (QoL) and state of health (SoH) [15–17] with DHI scores and found strong correlations between these measures and DHI scores. In contrast, no significant correlations were found between DHI scores and vestibular function tests such as posturography or vestibular ocular reflex tests [18]. The latter result is surprising as an uncompensated UVD might have been expected to influence dizziness [19, 20].

In view of these multiple associations with dizziness, we extended our investigation of the effects of an intervention program for patients with dizziness [11]. To do this, we performed multivariate stepwise regression analyses in order to estimate the associations of various predictor measures with the impact of dizziness as measured with the global DHI score. The predictor measures used were psychometric measures, as scored in the Brief Symptom Inventory (BSI) [21], impaired QoL, and perceived SoH. Finally, we examined the strengths of these associations for the selected predictor measures on emotional, functional and physical aspects of daily living as estimated by the DHI sub-scores. Because of the reported lack of correlation between balance deficits and DHI scores [18], we divided our study population into two groups, those with and without a balance deficit as determined by stance and gait tests in order to confirm this lack of correlation specifically in a group of patients with range of balance deficits without a “floor effect”. Our working hypotheses were [1] that psychometric measures from the BSI would be correlated with the emotional sub-score of the DHI and, [2] that QoL and perceived SoH would be correlated with the functional sub-score of the DHI. Further, that these relationships would be independent of whether a balance deficit existed, or not, as measured during stance and gait tests with body mounted gyroscopes.

## Methods

### Setting

This study was carried out at the Departments of Psychosomatic Medicine and ORL, University Hospital Basel, Switzerland. Data was used from patients with persistent dizziness who were treated with a multimodal intervention program, as described briefly below and in more detail in our previous publication [11]. A combination of cognitive behaviour therapy, balance training, and psychoeducation was used [11]. For the current retrospective study, patients were divided into 2 groups dependent on whether they had, pre-therapy, pathological balance control or not, with respect to healthy age-matched controls, in stance and gait tests [22, 23]. Patients provided informed consent for the use of their data for scientific purposes. The study (2014–026, Amendment 4) was approved by the local ethical committee responsible for the University Hospital Basel: Ethics Committee for North-Central Switzerland (German abbreviation, EKNZ). This report is based on regression analyses of an enhanced set of data collected from 40 patients between July 2013 and March 2017 when the study terminated. A previous publication [11] was based on repeated-measures analyses of variance from data collected from 32 patients within the same study between July 2013 and October 2015. The sample size of 40 was based on a power analysis (80%) that trends in phobic anxiety noted in our previous publication [11] would reach statistical significance (*p ≤ 0.05*).

### Study groups of subjects

The 40 subjects included in the study were accepted into our group therapy program if they suffered from persistent dizziness for at least 12 months, and their otoneurology tests results and a structured interview with a psychologist indicated that they fulfilled the inclusion criteria described below. All participants had been examined for vestibular deficits before the CBT treatment with a battery of video-oculographic oculomotor (optokinetic nystagmus and smooth pursuit eye tracking) and vestibular tests (caloric canal paresis and rotating chair) which tested for the presence of a central or peripheral vestibular deficit [24]. Patients were included if they fulfilled all of the following 7 inclusion/exclusion criteria: [i] no central vestibular deficit in oculomotor tests, [ii] no physical inability to participate in the physiotherapy exercises, [iii] no visual deficit affecting balance control, [iv] no evidence of a medium to severe form of depression established in the pre-therapy interview by a psychologist according to ICD-10 diagnostic criteria and supported by screening questions “During the past month, have you often been bothered by feeling down, depressed, or hopeless?” and “During the past month, have you often been bothered by little interest or pleasure in doing things?” [25, 26], [v] no indication of a lack of motivation or ability to cooperate with therapists and other patients in the group (e.g. lack of sufficient German language skills), [vi] no severe comorbid psychiatric disorders (e.g. schizophrenia), and [vii] no orthopaedic disorders that would have affected balance control. Most patients were transferred to our tertiary hospital from ear, nose and throat, neurology, and psychiatric practices as well as general practitioners in Basel and the area of north-west Switzerland.

As indicated in the table of demographic data (Table 1), patients’ dizziness was either constantly present or episodic. Additional demographic data is provided on education, marital status and psychiatric diagnosis in Table 2. The psychiatric diagnoses were determined by the author MS with support from DAS and WL. Differences between the groups are described in the results section.

**Table 1:** General, quantified balance deficit, dizziness and structural vestibular deficit data

	Quantified Balance Disorders (QBD) group	Dizziness Only (DO) group
N	20	20
Age mean ± SD	60.1 ± 9.9 years	45.6 ± 14.0 years
Gender M/F	8/12	6/14
Pathological BCI^a^	20/20	0/20
Duration of dizziness mean ± SD	3.5 ± 5.0 years	5.4 ± 7.7 years
Dizziness constant (fitting definition of PPPD^b^) or occurring in episodes.	12 constant/8 episodes	11 constant/9 episodes
Fear of falling	4/20	10/20
Deficits in VOR^c^ tests	15^d^/20	0/20
Unilateral peripheral vestibular deficit due to vestibular neuritis – not or over-compensated, 2 with active BPPN^e^, 4 with constant dizziness.	7/20	
Unilateral peripheral vestibular deficit due to vestibular neuritis – compensated. 6 with constant dizziness.	8/20	

^a^BCI stands for Balance Control Index, which is a combination of measurement values from stance and gait tests [22, 23]

^b^PPPD (persistent postural-perceptual dizziness) is defined according to the criteria of Staab et al. [27] and requires that dizziness be present for 15 of every consecutive 30 days

^c^VOR stands for vestibular ocular reflex. To be defined as a peripheral vestibular deficit the caloric paresis value had to be greater than 30%.To be defined as a not centrally compensated deficit, the rotating chair response asymmetry for yaw rotation accelerations of 20°/s^2^ towards and away from the deficit side had to be greater than 15%. To be defined as a centrally over-compensated, the deficit the rotating chair response asymmetry for rotation accelerations of 20°/s^2^ had to be less than − 15%. 30 and 15%, respectively, are the upper 95% limits of normal subjects for these tests

^d^The remaining 5 patients without deficits in VOR tests were assumed to have phobic postural vertigo, based on the criteria of Querner et al. [28] and Brandt et al. [29]. 2 had constant dizziness

^e^BPPN stands for benign proximal positioning nystagmus

**Table 2:** Education, marital status, and psychiatric diagnosis

	QBD group (*N* = 20)	DO group (*N* = 20)
**Maximum education level (N)**		
Obligatory	11	7
Vocational	6	7
Higher	3	6
**Marital status (N)**		
Married	11	10
Single	4	6
Divorced	5	4
**Psychiatric diagnoses (N)**		
Adjustment disorder	7	11^a^
Major depressive disorder, single episode, mild	4	5
Major depressive disorder, single episode, moderate	6	3^b^
Panic disorder without agoraphobia	3@	1
Mixed anxiety-depressive disorder	1	0

^a^ 3 with anxiety and depressive reactions, @ 1 with agoraphobia

^b^1 with panic attacks, 1 severe

### Interventions

The intervention in this study is described in detail in our previous publication [11]. It was designed by an interdisciplinary expert committee.

#### CBT

In brief, the eight 90 min sessions of weekly group therapy were offered to 10 persons per group on average (minimum 8 patients), and were always led by the same psychotherapist (author MS) specialised in CBT. Patient reports of situations in which dizziness occurred were analysed as part of a set of complaint-related behaviours.

#### Psychoeducation

As a typical element of cognitive behaviour therapy, medical and physiological information was offered (authors AWL and JHJA, respectively) about the multiple sources of chronic dizziness and the effect of anxiety on balance control.

#### VR

Patients practiced relaxation exercises and exercises in balance control under different conditions such as: standing on a foam surface, walking 8 tandem steps, walking with simultaneous head rotation.

### Measures

All subjects were asked to complete a set of psychological questionnaires provided in German and perform a sequence of stance and gait balance tests on average 7.3 (standard deviation (SD) 5.9) weeks before and 3.4 (SD 2.0) weeks after the intervention. The subjective impression of the impact of dizziness on daily life (primary outcome) was scored by the Dizziness Handicap Inventory (DHI) questionnaire, general health was captured by a quality of life (Qol) and state of health (SoH) questionnaires. Global and specific mental states (e.g. anxiety, obsessiveness, compulsiveness) were assessed using the Brief Symptom Inventory (BSI) described by its authors as “a brief psychological self-report symptom scale” [21] .

The *Dizziness Handicap Inventory (DHI)*, introduced by Jacobson and Newman [13], assesses the subjective impression of the impact of daily life impairments caused by dizziness. We used a German translation of the DHI, which was validated by Volz-Sidiropoulou et al. [14]. The DHI includes 25 items, has a global score and captures 3 aspects of impairment: emotional, functional and physical. Examples of questions for these 3 aspects are, respectively: “Because of your dizziness or unsteadiness problem, do you feel frustrated?”; “Because of your problem, do you have difficulty reading?”; “Do quick movements of your head increase your problem?” Participants could choose to answer in 3 ways: yes (score 4), sometimes (score 2), or no (score 0). Thus the maximum global score was 100.

The *EuroQol* questionnaire was introduced by EuroQol Group [30, 31] and measures the patient’s general state of health. We used the EQ-5D-3 L, a five-dimensional version with 3 levels of impairment. The validity of the German version used [31] was verified by Konerding et al. [32]. The five dimensions include mobility, self-care, usual activities, pain/discomfort and anxiety/depression, each captured with one item. The 3 levels of impairment are no problems scored 1, some problems scored 2, and extreme problems scored 3. The questionnaire includes a visual analogue scale (EQVAS), which captures the patient’s self-rated state of health (SoH) on a 20 cm vertical, visual analogue scale. There is an extensive body of literature to support the validity and reliability of the 3 L descriptive system, the EQVAS, and the 3 L index values in many conditions and populations [33]. The EQVAS scores are anchored on 100 equal to the best imaginable health and 0 equal to the worst imaginable health.

The *Brief Symptom Inventory (BSI)* was introduced by Derogatis and Melisaratos [21] and evaluates psychological distress and psychiatric disorders. The questionnaire has 53 items using a 5 point scale (0 not at all, 1, 2, 3 to 4 very strongly). In addition to a global score (GSI) based on all items, the BSI measures 9 aspects with sub-scores: somatization, obsessive-impulsive, interpersonal sensitivity, depression, anxiety, hostility, phobic anxiety, paranoid ideation and psychoticism. According to Derogatis and Melisaratos [21], the BSI has good internal consistence showing an average rating above α = 0.7 for the scales. The test-retest reliability ranges from 0.68 to 0.87. We used Franke’s German version of the BSI [34]. The reliability and validity of this German version was verified by Geisheim et al. [35].

*Balance Control Index (BCI):* We used the same techniques described previously [22, 23, 36] to measure balance control during stance and gait tasks and to establish whether balance control was within normal limits or not. For this purpose, lower trunk sway was recorded with a gyroscope system (SwayStar™ Balance Int. Innovations, Switzerland). The stance and gait tests comprised 14 tasks: [tasks 1 & 2] standing on two legs with eyes open and with eyes closed; [tasks 3 & 4] standing on one leg with eyes open and with eyes closed; [task 5] walking eight tandem steps while looking at the feet; [tasks 6 & 7] standing on a foam on two legs with eyes open and with eyes closed; [task 8] standing on a foam on one leg with eyes open; [task 9] walking eight tandem steps on foam while looking at the feet; [tasks 10, 11 & 12] walking three meters while flexing and extending the neck, while rotating the head from side-to-side, or with eyes closed; [task 13] walking up and down a set of stairs with 2 steps up and 2 down; and [task 14] walking over 4 low barriers 24 cm high and spaced 1 m apart. The test results were combined and used to calculate a *Balance Control Index (BCI)* [22, 23] for every patient. Every BCI was then compared to age-matched normal reference values [23]. A patient was assumed to have an objectively determined balance deficit if the patients’ BCI was greater than the normal age-matched upper 95% value. Based on this BCI criterion 2 groups of 20 patients were defined: those with a so-defined balance deficit whom we termed as patients with an “objectively” quantified balance deficit (QBD) and patients with normal BCI values who were classified as having normal balance control and dizziness only (DO).

Within each group of patients (QBD and DO), we noted (see Table 1) whether dizziness was constantly present or not, the presence or absence of a structural vestibular deficit, and the presence of phobic postural vertigo defined according to the criteria of Querner et al. [28] and Brandt et al. [29]. For 11 of the 20 DO patients, dizziness was constant (occurring on 15 days or more each month, according to the accepted criterion [27]) and therefore they could be diagnosed as having persistent postural-perceptual dizziness (PPPD). For the other 9 patients the dizziness consisted of reoccurring episodes of dizziness (less than 15 days per month) (see Table 1). Slightly more of the QBD patients (12 of 20) had constant dizziness. As indicated in Table 1, 15 of the QBD and none of the DO patients had a chronic structural unilateral peripheral vestibular loss as determined by caloric tests [24], that is, a canal paresis values was greater than 30% [24], or by the presence of benign proximal positioning nystagmus (BPPN). Six of these patients (see Table 1) had a vestibular deficit which was not centrally compensated based on their rotating chair response asymmetries being greater than 15% [24]. One patient had an over-compensated vestibular deficit because the patient’s rotating chair asymmetry was less than − 15% [24]. In all these patients the vestibular loss was assumed to be due to vestibular neuritis based on the clinical signs on acute clinical manifestation and subsequent caloric and rotating chair tests. Two patients had benign paroxysmal positioning nystagmus (BPPN), one in addition an uncompensated peripheral vestibular loss. The other 5 of the QBD patients and none of the DO patients had phobic postural vertigo defined according to the criteria of Querner et al. [28] and Brandt et al. [29]. That is, the patients had pathological balance control, as scored with the BCI, and performed more difficult balance tasks with better scores than easier balance tasks of the same type, e.g. walking 8 tandem steps on foam compared to walking on a firm support surface.

### Statistical analysis

A repeated-measures analysis of variance was performed with IBM SPSS Statistics to estimate possible differences between the QBD and DO groups mean values pre- and post-therapy. Post-hoc paired t-tests were performed between pre- to post-therapy values that were calculated across both groups as well as individually for each group. The level of significance was set at 0.05 taking into account Bonferroni corrections.

Several linear regression analyses were performed separately for each group using Matlab (Statistical Toolbox (R2018b), The MathWorks, Inc.). First, single regressions were performed between the DHI scores and the 8 BSI sub-scores, the BCI, QoL and the perceived state of health (SoH) scores. Then, a step-wise multivariate linear regression analysis was performed in two stages. The first stage involved a step-wise regression between DHI scores and all 8 BSI scores. The second stage involved including the BCI, QoL and perceived SoH scores in the step-wise regression. The step-wise regression technique proceeded by selecting the independent variable having the highest regression coefficient with DHI scores to enter the multivariate regression. Then in the second step, the covariation accounted by first selected variable is removed from the group of remaining variables to be entered before the next regression variable with the highest regression coefficient to DHI scores is sought again. This procedure is then repeated until none of the to-be-entered variables has a significant regression to enter. We checked for adequate power to the multi-variate regressions, given the number of entered variables using G*Power (Dept of Psychology, University of Düsseldorf, https://is.gd/d3mvDd), with an effect size of R^2^/(1-R^2^), where R is the regression coefficient. All multi-variate regressions had a power of 0.95 given a test sample of less than 20 subjects. Finally, regressions were also performed between each DHI sub-score (emotional, functional and physical) and those predictor variables chosen to enter the stepwise multivariate regression analysis. Our primary outcome, the DHI, and variables entering into the stepwise regression analysis were tested for a normal distribution using Kolmogorov-Smirnov tests. The DHI scores, had the highest *p* values for these tests (0.8 and 0.9 for DO and QBD groups, respectively). We used a parametric repeated-measures analysis of variance and linear regression techniques because the only variable that was not normally distributed (*p* = 0.01) according to these tests was phobic anxiety for the dizziness only (DO) group, probably due to a floor effect post-therapy.

## Results

This report is based on data from 40 patients (14 (35%) male) including data from 32 patients who featured in our previous publication [11]. The DO group comprised 20 patients who had a mean age of 45.6 (standard deviation (SD) 14.0) years. The QBD with dizziness group had an equal number of patients [20] with a mean age of 60.1 (SD 9.9) years. There was a significant difference between the mean age of each group (*p < 0.001*). Obligatory school education (9 years) was completed by slightly more of the QBD group. Psychiatric diagnoses were not different between the groups (Table 2). The majority of the patients had an adjustment or depressive disorder (Table 2). Marital status was also similar in both groups (Table 2).

Values of the primary outcome, DHI score, were used as predicted values for the regressions reported here. Values of secondary measures, balance control scores and psychological outcomes from the BSI questionnaire, were used as predictor measures in the multivariate stepwise regression analysis of this report. Means of these measures were reported pre- and post-therapy for 32 subjects in our previous report [11]. Because knowledge of the means of these values aids interpretation of the current regression analysis, we report briefly below on these means for 40 subjects. Unless otherwise noted, there were no substantial changes in the significance of the differences in these means pre- compared to post-therapy with more subjects included in the DO and QBD groups, i.e. between the previous [11] results and those reported here.

### Dizziness handicap inventory (DHI) outcomes

There was no substantial difference between the number of patients in each group with constant or episodic types of dizziness. Constant dizziness (we used the definition of Staab et al. [27], that is, being present for 15 or more days of each month) was reported by 11 patients of the DO group and 12 of the QBD group. The remainder, 9 DO patients and 8 QBD patients reported reoccurring episodes of dizziness. Dizziness had lasted for more than 1 year pre-therapy for all patients.

**Fig. 1:**
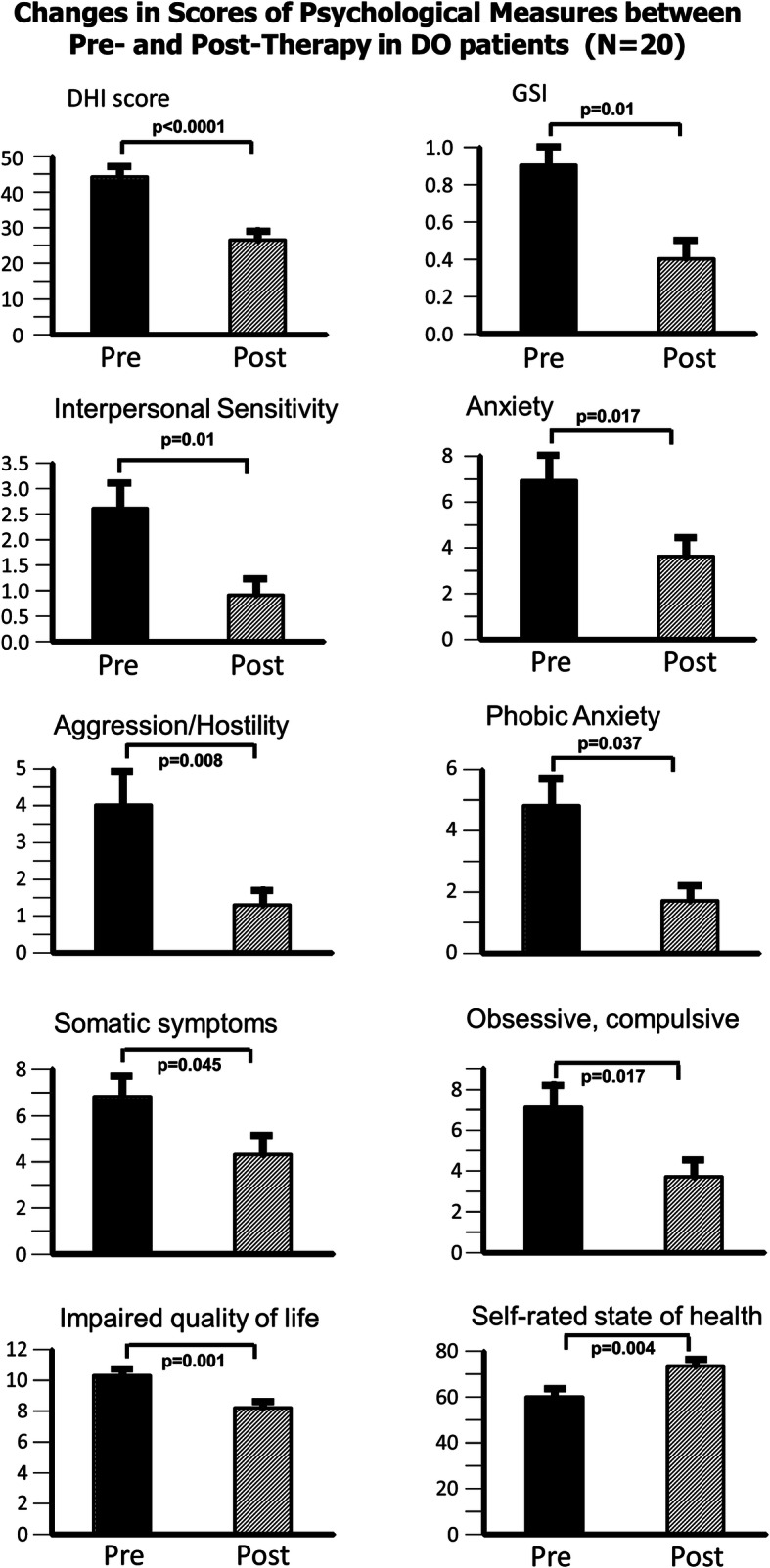
Mean scores pre- and post-therapy for patients with dizziness only (DO). Illustrated are the means pre- and post-therapy of the primary outcome Dizziness Handicap Inventory (DHI) score, Global Score Index (GSI- the sum of the scores from the answers to all questions divided by the number of questions in the BSI questionnaire), GSI psychometric sub-scores (obtained from the BSI questionnaire), perceived impaired quality of life, and perceived state of health. The column heights in each panel represent the mean values and the vertical bars on the columns, the standard error of the mean. A horizontal bar with a probability (p) value indicates a significant difference between the means. The DHI has a maximum possible score of 100. The changes in the mean Balance Control Index (BCI) from pre- to post-therapy for patients with dizziness only (not displayed) were not significant. The BCI combines of stance and gait balance tests results (see Hegeman et al. [23]) in an optimal form to aid identification of patients with peripheral vestibular deficits [22] when compared to normal controls

**Fig. 2:**
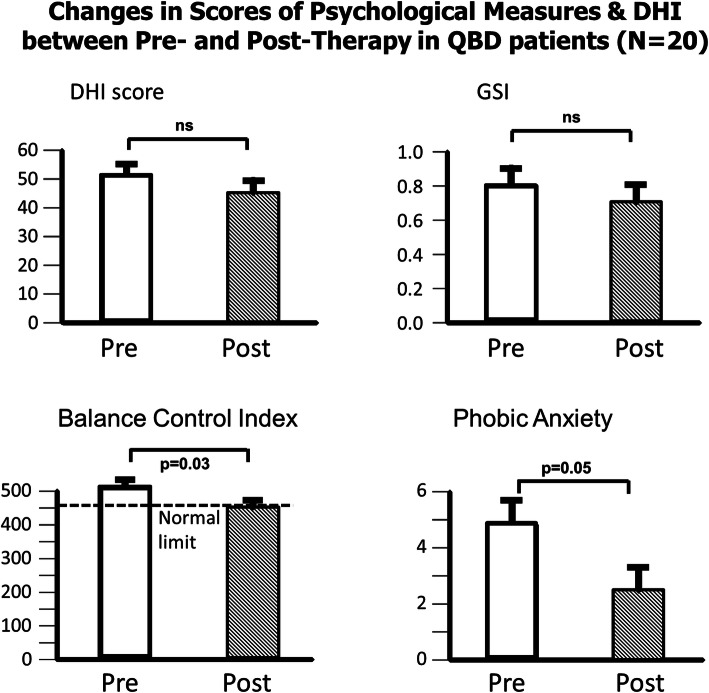
Mean scores pre- and post-therapy for patients with dizziness and a quantified balance deficit (QBD) for the primary outcome measure Dizziness Handicap Inventory (DHI) score, and secondary measures: the Global Score Index (GSI), phobic anxiety psychometric sub-score (obtained from the BSI questionnaire), and BCI values. As in Fig. 1, the column heights in each panel equal the mean values and the vertical bars on the columns, the standard error of the mean. A horizontal bar with a probability (p) value indicates the significant difference between the means if present. Only the changes, pre- to post-therapy, in the mean Balance Control Index (BCI) and phobic anxiety were significant. Other psychometric scores of QBD patients were not changed post therapy. The normal upper limit for the BCI is indicated by the dashed line in the lower left panel

The changes in the global DHI scores pre- to post-therapy for DO and QBD patients are shown in the upper left column plots of Figs. 1 and 2, respectively. Patients subjective assessment of the impact of dizziness, as scored with the DHI, improved significantly (*p < 0.0001*) post-therapy in the DO group. There was no change, post-therapy, for the QBD group. Pre-therapy, DHI scores did not differ between the two groups. As the mean DHI score of the DO group was greater than 30, pre-therapy, but less than 30 post-therapy, this change implied an average change from a medium to a minor, self-assessed, balance impairment. There are 3 DHI sub-scores - emotional, functional and physical impairment. For the DO patients, all 3 sub-scores showed the same pattern as observed for the global DHI score with significant improvement post-therapy (*p ≤ 0.003*). These post-therapy sub-scores were then lower than those of the QBD group (*p ≤ 0.05*).

### Balance control outcomes

The BCI score was used to assign patients to one of the 2 groups, DO or QBD. Therefore, pre-therapy, each group’s mean BCI values were significantly different (*p < < 0.001*). As the mean ages of the patients in each group were different (45.6 versus 60.1 years), the question arises if the difference in BCI scores can be explained by age. There are, however, no changes in mean BCI values in healthy subjects between the ages of 40 and 65 years [23]. There was no improvement in BCI values in the DO group post-therapy. The values in the DO group were normal, however, pre-therapy. Post-therapy, improved balance was noted for the QBD group (*p = 0.03*) bringing the mean BCI value equal to the upper 95% limit of normal (Fig. 2).

### Psychological outcomes

Prior to CBT, BSI questionnaire scores were not significantly different between the groups (*p > 0.05*). However the scores were different from those of healthy normal subjects. Mean values of the BSI for healthy controls are provided by Franke [37]. After therapy, the global psychological score (GSI) and all sub-scores showed a significant improvement for the DO group (*p = 0.01*). The QBD group had no significant improvement in the GSI scores post-therapy. This pattern of results was similar to that of primary outcome measure, the global DHI score (see Figs. 1 and 2). The most significant improvements in secondary outcomes (see Fig. 1) observed in the DO group were for the values of the BSI sub-scores aggression/hostility (*p = 0.008*), interpersonal sensitivity (*p = 0.01*), obsessive/compulsive (*p = 0.017*) and anxiety (*p = 0.017*). The QBD group did not have a significant improvement for the BSI global score (Fig. 2) post-therapy. The phobic anxiety sub-score was the only BSI sub-score that improved for this group (Fig. 2) being more significant (*p = 0.05*) with 20 patients compared to 16 (*p = 0.16*).

### Perceived quality of life (QoL) and state of health (SoH)

Similar to the GSI psychometric scores, these two measures improved for the DO group (see lower panels Fig. 1), but not for the QBD group. Thus QoL (*p = 0.001*) and SoH (*p = 0.004*) improved after therapy for DO subjects indicating that these subjects perceived their health more positively post-therapy.

### Regression analyses

Because several psychometric variables, QoL, and perceived SoH improved for the DO group (Fig. 1) but only showed trends for the QBD group, the question arises which variables have the greatest association with the DHI and its sub-scores within each subject group, and whether these possibly influencing variables are common between groups. The question also arises if balance control, as measured with the BCI score, is also correlated with the dizziness handicap (DHI). We attempted to answer these questions using a combination of simple and stepwise multi-variate linear regressions, yielding a final model including all predictive variables for the DO and QBD groups (see Tables 3 and 4).

**Table 3:**
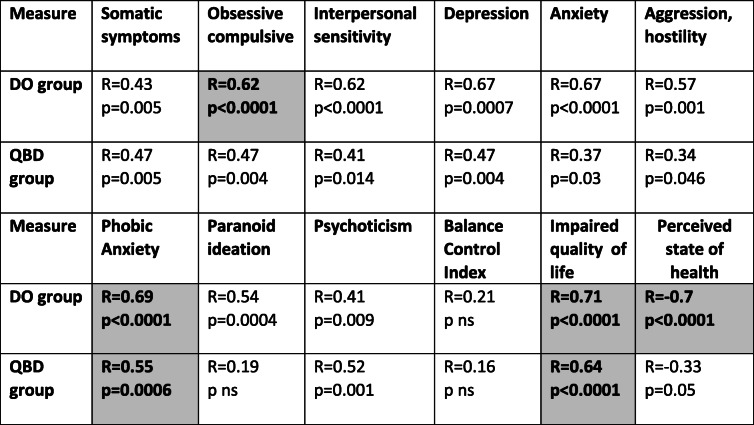
Regression coefficients (R) and significance (p) for dizziness only (DO) and quantified balance deficit (QBD) groups between measures of psychometric state (BSI), balance deficit (BCI), impaired quality of life, and state of health when regressed with DHI scores. ns stands for not significant (*p* > 0.05). The regression coefficients are for a regression of a single secondary score measure and the DHI score. The measures in bold text and highlighted grey are those measures which entered the multi-variable regressions shown in the lower and upper parts of Figs. 3 and 4. The BCI score was not used for the multivariate regression as there was no significant correlation to the DHI score for both the DO and QBD groups. All other measures depicted in the table could enter the multi-variable regression

**Table 4:**
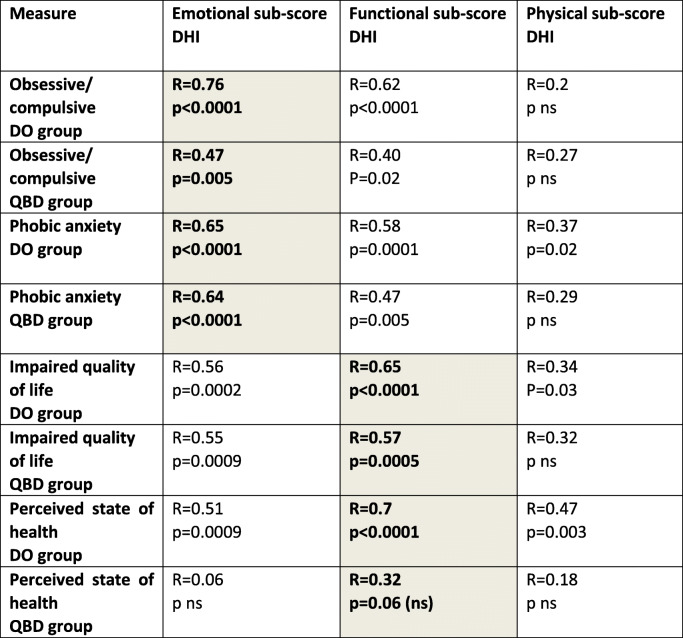
Regression coefficients (R) and significance (p) for DO and QBD groups between measures entering the multivariate regressions depicted in Figs. 3 and 4: phobic anxiety and obsessive/compulsive behavior, impaired quality of life, state of health when regressed with the DHI sub-scores, emotional, functional, and physical. ns stands for not significant (*p* > 0.05). The regression coefficients are for a regression of a single measure and the DHI sub-score. The regressions in bold text and highlighted grey are the highest regressions across DHI sub-scores

Table 3 lists the correlation coefficients R and associated significance for each secondary variable compared to the DHI scores separately for each patient group. The weakest correlations with DHI scores of both groups were observed for the BCI score. This correlation did not increase using any of the DHI sub-scores instead of the global DHI score. The strongest correlations in both groups were for the BSI sub-scores phobic anxiety and obsessive/compulsive behaviour (Table 3). The other two secondary variables QoL and SoH, QoL showed the strongest correlations to DHI (Table 3).

**Fig. 3:**
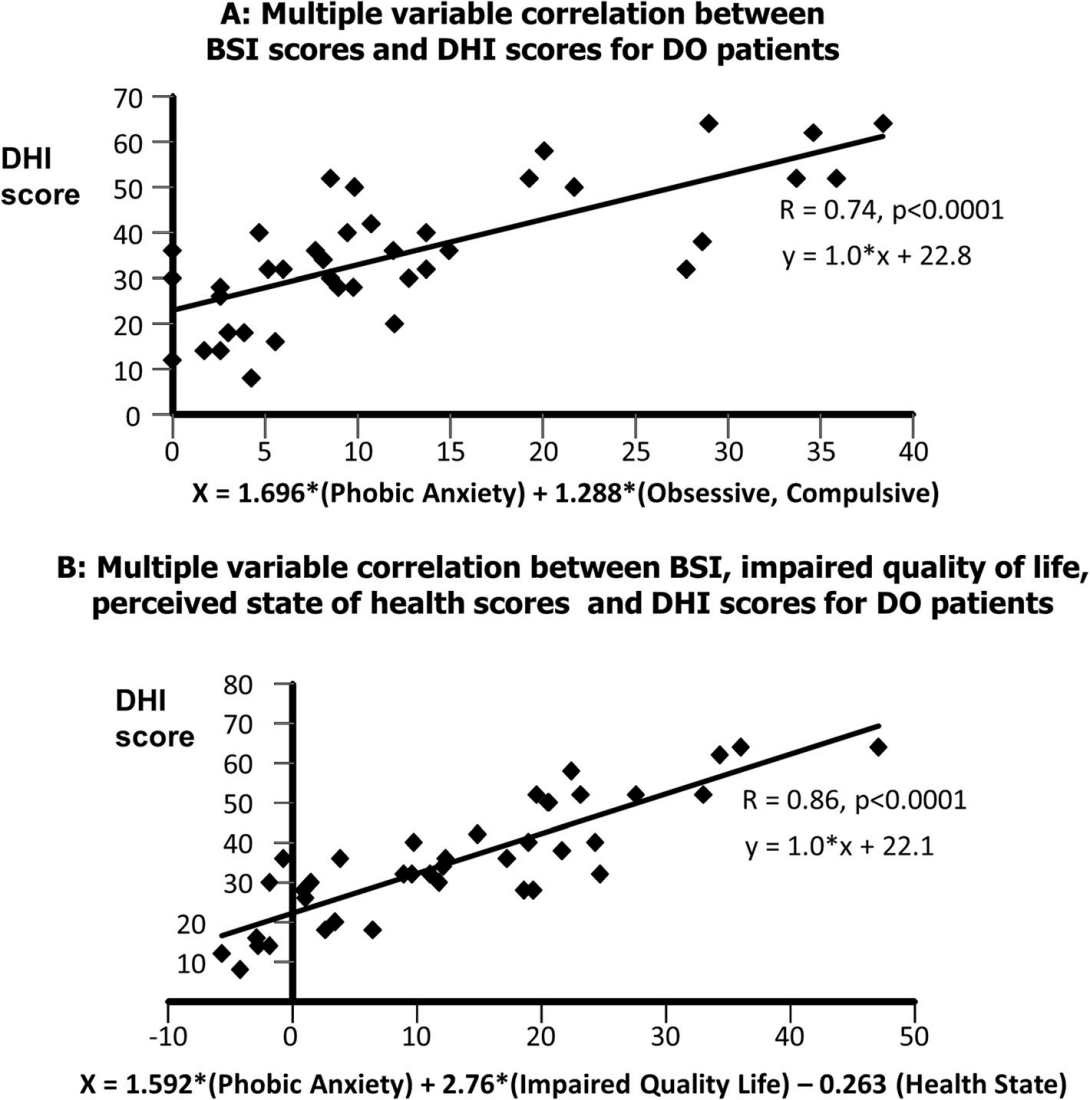
**a** Regression analysis results of stepwise multivariate correlation analysis between psychometric BSI scores and the DHI scores for the DO patients and **b** (lower plot) with impaired quality of life and health state included among the possible to-be-entered variables. Both regressions are significant. The coefficients applied to the selected variables are taken from the multivariate regression analysis. The regression in **b** shows a higher correlation (R = 0.86 vs. 0.74 in **a**). The BCI was not selected to enter the regression from the list of to-be-entered variables as the correlation BCI scores with DHI scores was not significant – see Table 3

**Fig. 4:**
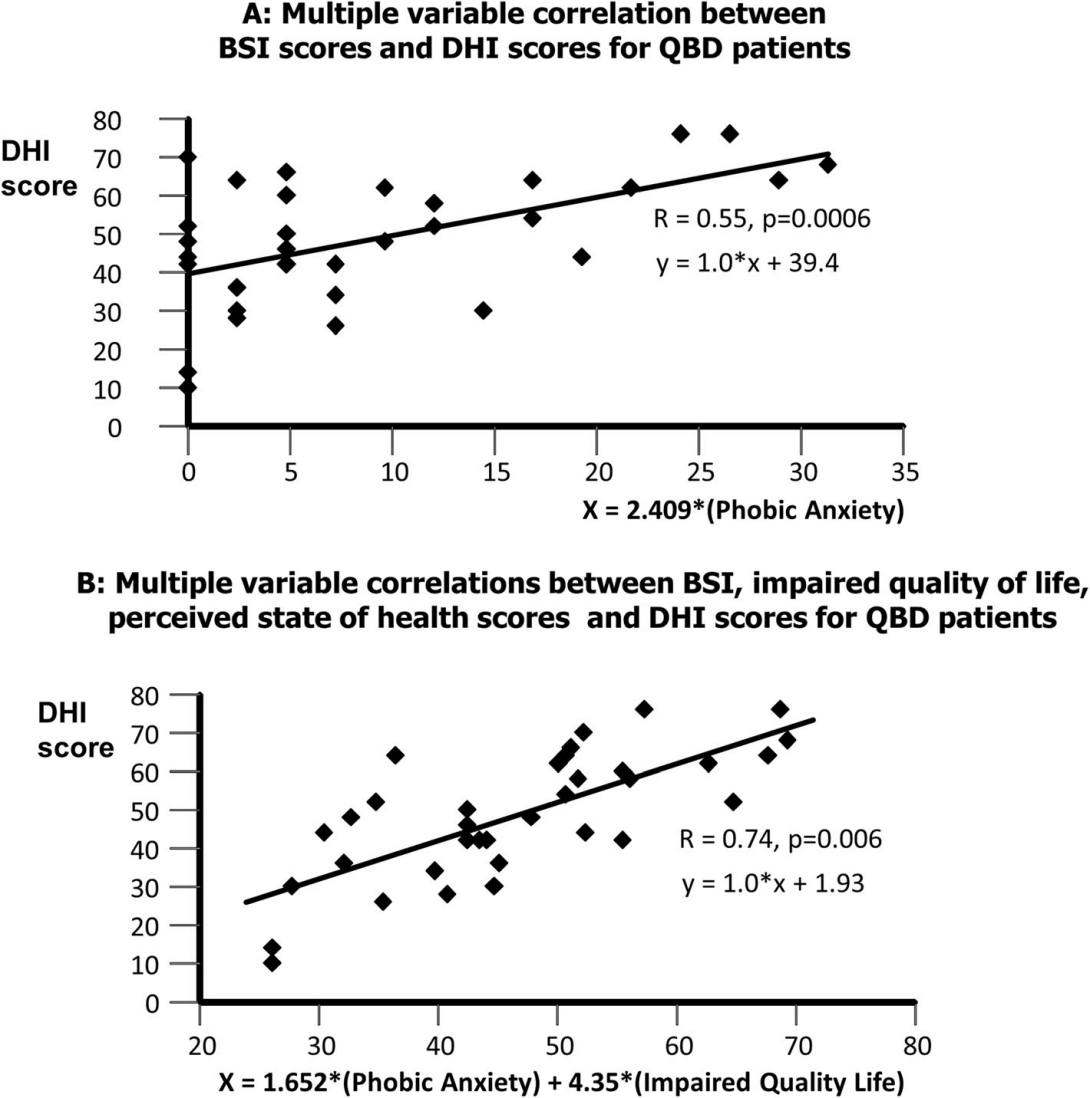
**a** Results of stepwise multivariate correlation analysis between psychometric BSI scores and the DHI scores for the QBD patients and **b** (lower plot) with impaired quality of life and health state included among the to-be-entered variables. Both regressions are significant. The coefficients applied to the selected variables are taken from the regression analysis. For **a**, only one variable, phobic anxiety entered the regression analysis. The regression in **b** shows a higher correlation (R = 0.74 vs. 0.55 in **a**). The BCI was not included in the list of to-be-entered variables as the correlation with DHI values was not significant – see Table 3

When stepwise regression analyses were performed with all BSI sub-scores, phobic anxiety was selected for both groups as the best correlated variable with DHI scores. For the DO group, obsessive/compulsive behaviour was selected in addition. The resulting correlations are shown in the upper part of Figs. 3 and 4. These correlations indicate that 30% of the variance in QBD patients’ DHI scores can be accounted for by phobic anxiety. Further that 55% of the variance for DO patients’ DHI scores can be accounted for by phobic anxiety and obsessive/compulsive behaviour. These figures increase to 55 and 74%, respectively, when QoL entered the QBD group regression and when both QoL and SoH entered the DO group regression (see lower parts Figs. 3 and 4).

When the correlations of the four secondary variables selected by the multivariate stepwise regression analysis to each DHI sub-score were examined, the highest correlations for both groups were between emotional DHI sub-scores and phobic anxiety and obsessive/compulsive behaviour (Table 4). QoL and SoH were best correlated with the functional DHI sub-score. While correlation does not imply a direct cause and effect, it is interesting that the patterns established with this regression analysis are common for both dizziness groups. Possible implications are discussed below.

## Discussion

The aim of this study was to investigate the strength of the associations between patients’ perceived state of health, perceived impaired quality of life, balance control, and mental states (BSI scores) with the impact of their perceived dizziness on daily living as measured by Dizziness Handicap Inventory (DHI) scores. For this purpose we examined the strengths of regressions of these to be associated measures to DHI scores. Despite differences in balance control abilities between patients with dizziness only (DO) and patients with dizziness and quantified balance control deficits (QBD), our major finding was to show for both of these patient groups, common relationships of the patients’ perceived impact of dizziness, as recorded by DHI scores, with BSI psychometric measures, perceived impaired QoL scores, and perceived SoH measures. In QBD patients, phobic anxiety and QoL accounted for 55% of the variance in DHI scores. In DO patients, 75% of the variance of DHI scores was accounted for by these measures with the addition of obsessive/compulsive behaviour and SoH scores. Subsequent regression analyses with these variables indicated, as hypothesized, that the psychometric measures were predominantly associated with the emotional DHI sub-scores (Table 4). The QoL and SoH measures had a major association with the functional DHI sub-scores (Table 4).

In QBD patients, a lower correlation with DHI scores was found for psychometric, QoL and SoH measures. This is presumably the result of the weaker effect CBT combined with VR had on these measures for QBD patients compared to DO patients. In this respect, recent evidence reported by Decker et al. [38] may have had an influence on our results. These authors provided evidence that a normally operating peripheral vestibular system is required for the development of anxiety related to dizziness, possibly for reasons we describe below. However, we did not note a difference in pre-therapy anxiety scores between the 2 groups of patient even though most of the QBD group had a unilateral peripheral vestibular deficit. Our findings confirm results of our earlier report concerning psychometric scores with fewer subjects [11]. In contrast to our earlier report however, the current regression analysis provides stronger evidence that both groups of patients benefitted especially from a marked reduction of phobic anxiety with CBT. This aspect is predominantly addressed in the cognitive behavioural part of our intervention even though we cannot rule out that psycho-education contributed as well, by providing realistic instead of catastrophic information on the causes of dizziness to patients. Surprisingly, an improvement in balance control did not translate into an improvement in the perceived dizziness scores, even though balance control clearly improved post-therapy in the QBD group (see Fig. 2). Thus a primary open question concerns the approach to be taken with VR in future studies. Possibly, the effect of CBT on DHI scores should be examined with and without VR.

The lack of correlation between balance (BCI) and the perceived impact of dizziness (DHI) scores appears at first sight to be contradictory, especially as these measures are correlated for patients with central lesions such as multiple sclerosis [39] and mild traumatic brain injury [40]. However, others have also reported on a lack of correlation with DHI scores for patients with unilateral peripheral vestibular loss (UVL) [41–43]. The majority of our QBD patients had a UVL (Table 1). Furthermore, in an extensive study of several hundred patients no correlation was found between DHI scores and vestibular ocular reflex gains, vestibular spinal reflex amplitudes (Vemps) or posturography scores in patients with UVL [18]. This lack of correlation may be due to differences between balance test situations and the questions asked as part of the physical DHI sub-score. For example, none of the questions in the DHI questionnaire asks about standing with eyes closed or on leg with eyes open which are typically tested during examinations of postural control.

The improvement in balance control of the QBD group is presumably the result of the VR component of our intervention. This element of the multimodal CBT intervention program has been shown to work in older individuals too [44]. However, there was a noteworthy mean difference in age (15 years) between the two groups of patients we investigated, which is a potential limitation of this study. While age differences may not have affected balance control directly [23], this difference could have affected the ability to learn and benefit from the CBT techniques leading to a weaker effect on psychometric, QoL and SoH measures for the older group of QBD patients. Even though elderly persons, when treated for generalised anxiety disorder with CBT, show no differences with respect to working-age persons, the effect size was lower for the elderly [45]. Thus, the difference in age could also explain the 20% decrease in variance in DHI explained by the psychometric, QoL and SoH measures in our older QBD group compared to the younger DO group.

Our findings on the significant correlations of impaired QoL and perceived SoH with the DHI scores of patients with dizziness corroborate those of other studies [15–17]. In our study including these variables in correlations with psychometric measures led to a further 23 and 25% increase in explanation of the variance in DHI scores for DO and QBD patients, respectively. Our results support those of Limburg et al. [12] by showing that variables such as health anxiety or health-related cognitions are relevant predictors differentiating therapy outcomes. These findings raise the question whether CBT could be enriched by emphasising these subjective accounts of personal health related QoL in addition to focussing on phobic anxiety and obsessive/compulsive behaviour. The emphasis on those aspects during CBT has been shown to be effective in the treatment of somatoform disorders [46].

An aspect we have not taken into account is the history of traumatic aspects of vestibular deficits our patients experienced in terms of embodied anxiety (continuing or newly triggered), contributing to our results. Phobic anxiety components could be interpreted as potentially trauma related stress symptoms including avoidance aspects. In this respect, Radziej et al. 2015 [47] postulated that exposure to trauma and symptoms of posttraumatic stress can contribute to symptom severity and handicap experienced by patients with vestibular symptoms irrespective of their original cause, most likely serving as predisposing, modulating or perpetuating factors.

As far as the focus of CBT is concerned we would also argue that considerable attention should be given to the influence of anxiety on perceived balance stability in both groups of patients. This focus of a CBT intervention which aims to decrease fear of bodily sensations and cognitions, and symptom-related beliefs about these symptoms is presumably useful as these fears have been shown to play a mediating role in the relationship between psychopathology and dizziness/vertigo symptoms [47]. This recommendation is also based on the known effects of anxiety on vestibular and proprioceptive sensory signals contributing to balance control. For example, chronic anxiety disorder is known to influence vestibular ocular reflex gains [48, 49]. Crucial information for those treating patients with phobic anxiety is that state anxiety induced in normal subjects by raising them to a height of 3.2 m and asking them to stand on the edge of the platform leads to increases in the gains of vestibular spinal and proprioceptive reflexes [50, 51]. When normal subjects were asked to register their perceived body sway, this perception was larger at the height of 3.2 m compared to at ground level for the same amplitude of induced trunk sway [52]. Presumably, the changes in anxiety related perceived increases in sway amplitude are the result of increased sensory gains with anxiety and lead to faster responses for the same amplitude of sway. A neural substrate exists for these gain changes in the form of neural centres activated by anxiety synapsing on brainstem vestibular nuclei [53]. These physiological mechanisms describing the effects of anxiety on sway perception thereby inducing dizziness are applicable to subjects with normal balance control as were the subjects in the DO group. For subjects with a peripheral vestibular deficit, as the majority of our QBD group were, vestibular gains are decreased and presumably also the influence of anxiety on these gains as Decker et al. [38] suggested.

## Conclusions

The major finding of this study was to demonstrate for two different groups of patients with dizziness, one with and the other without accompanying balance deficits, similar relationships of the patients’ perceived impact of dizziness, as recorded by DHI scores, to phobic anxiety and perceived impaired QoL scores. Our results also support recent evidence indicating that DHI scores are not correlated with deficits in coexisting balance control measures. This report’s regression analysis provides evidence that reducing phobic state anxiety and obsessiveness/compulsiveness may be a relevant focus for CBT combined with VR in order to improve emotional aspects of dizziness. Similarly, targeting perceived SoH and QoL may help to improve functional aspects of dizziness. We believe these new findings will have important implications for future cognitive-behavioral therapy treatment of patients with dizziness. These results also provide insights into measures best to use when screening for patients likely to develop chronic dizziness while it is still developing.

## Data Availability

Is available and will be provided by the authors on request.

## Abbreviations

BCI: Balance control index
BPPN: Benign proximal positioning nystagmus
BSI: Brief symptom inventory
CBT: Cognitive behavioral therapy
DHI: Dizziness handicap inventory
DO: Dizziness only
ICD: International classification of diseases
N: Number of
QBD: Quantified balance deficit
QoL: Quality of life
*p*: probability
PPPD: Persistent postural-perceptual dizziness
R: Regression correlation coefficient
SD: Standard deviation
SoH: State of health
UVD: Unilateral peripheral vestibular deficit
VR: Vestibular rehabilitation
